# High-cutoff hemodialysis in multiple myeloma patients with acute kidney injury

**DOI:** 10.3389/fonc.2022.1024133

**Published:** 2022-10-26

**Authors:** Yan Xing, Jipeng Yan, Zixian Yu, Jin Zhao, Yuwei Wang, Xiayin Li, Yunlong Qin, Shiren Sun

**Affiliations:** Department of Nephrology, Xijing Hospital, Fourth Military Medical University, Xi’an, Shaanxi, China

**Keywords:** multiple myeloma, acute kidney injury, tubular nephropathy, high-cutoff hemodialysis, high-cutoff membrane

## Abstract

Multiple myeloma (MM), an incurable hematological malignancy with clonal proliferation of plasma cells, is mainly characterized by excessive production of monoclonal immunoglobulins and free light chains (FLCs). Kidney injury is one of the main clinical manifestations and is also a significant predictor of the prognosis of symptomatic MM patients, especially those who require dialysis-supported treatment. Overproduction of FLCs is the trigger for kidney injury, as they can induce the transcription of inflammatory and profibrotic cytokines in the proximal tubule and bind to Tamm–Horsfall protein in the distal tubules to form casts that obstruct the tubules, leading to kidney injury and even renal fibrosis. In addition to traditional antimyeloma treatment, high-cutoff hemodialysis (HCO-HD), which can effectively remove FLCs *in vitro*, has attracted much attention in recent years. Due to its greater membrane pore size, it has significant advantages in removing larger molecules and can be applied in rhabdomyolysis, sepsis, and even myeloma cast nephropathy. However, mounting questions have recently been raised regarding whether HCO-HD can truly provide clinical benefits in MM patients with acute kidney injury (AKI). Therefore, in this study, we discussed the pathological causes of AKI secondary to MM and summarized the current situation of HCO-HD in MM patients compared with other available extracorporeal techniques. In addition, pivotal clinical trials that reflect the ability of the clearance of FLCs and the side effects of HCO-HD are highlighted, and the relevant protocol of HCO-HD is also provided to assist clinicians in decision-making.

## Introduction

Multiple myeloma (MM) is a malignant proliferative tumor of plasma cells, and is the second most common hematological malignancy (1), accounting for 13% of malignant hematological diseases and 1% of all malignancies (2, 3). MM affects mostly elderly men aged approximately 65 years (4), with an age-standardized incidence rate of 2.1/100,000 in 2016 worldwide (5). MM is two to three times more common among people of African descent than among Caucasian people, while it is less common among Asian and Hispanic people (6, 7). With improved diagnostic procedures and increased clinical knowledge regarding this disease, the number of people suffering from MM is steadily increasing. Hypercalcemia, kidney injury, anemia, and bone lesions are the most common symptoms of symptomatic MM (3, 8). Kidney injury has previously been proven to be an important risk factor that has a direct impact on patient survival (9–11). According to studies on symptomatic MM, approximately 20%–50% of patients who present with symptoms will develop kidney injury (2, 12–15), 12%–20% of patients will develop acute kidney injury (AKI), and approximately 10% of patients with AKI will eventually require dialysis treatment (16, 17). Previous studies have shown that 70%–90% of dialysis-supported patients with AKI secondary to MM develop myeloma cast nephropathy (MCN) due to the significant production of monoclonal free light chains (FLCs) by malignant plasma cells (18, 19). Therefore, effective clearance of FLCs is particularly imperative in these patients. With the advent of chemotherapy drugs such as proteasome inhibitors, immunomodulatory imide drugs and monoclonal antibodies, the production of FLCs has been suppressed, and the prognosis for MM has steadily improved. Although chemotherapy, which can inhibit the formation of FLCs, is the cornerstone, extracorporeal techniques that can facilitate the removal of FLCs are likewise receiving much interest.

## Extracorporeal techniques

Solute and water can be removed by using different mass separation mechanisms, which are diffusion, convection, and adsorption (20, 21). Plasma exchange (PE) is another purification method in which a patient’s plasma and blood cells are separated and disease-causing plasma or hazardous substances are filtered out (22). Conventional hemodialysis is diffusion-based and removes small molecules such as urea nitrogen and serum creatinine. For middle molecules like β2-microglobulin, high-flux hemodialysis (HF-HD) has more power to remove them than conventional hemodialysis. However, due to the size of the membrane pores, HF-HD can only remove molecules of approximately 10–20 kDa, while the molecular weights for the κ and λ chains of FLCs are 22.5 and 45 kDa, respectively. Therefore, HF-HD is theoretically ineffective in removing FLCs from the blood, but some FLCs can be removed due to the nonuniformity in pore size (23). With advancements in dialysis mode and membrane technology, hemodiafiltration with ultrafiltrate regeneration by adsorption in resin (SUPRA-HFR) based on diffusion, convection, and adsorption (24); high-cutoff (HCO) membranes and medium-cutoff (MCO) membranes with larger membrane pores (25); and polymethylmethacrylate (PMMA) membranes with powerful adsorption have been developed (26). An HCO membrane with pores ranging from 0.008 to 0.01 μm and a 50- to 60-kDa cutoff in blood, which can be used in rhabdomyolysis and sepsis, is also effective in removing FLCs from the blood and can thus be used in MM. The size of the membrane area is the fundamental difference between the two frequently adopted types of HCO filters, HCO1100 and Theralite^®^, which is 1.1 m^2^ for HCO1100 and 2.1 m^2^ for Theralite^®^. HCO1100 can be used in series to yield a better effect as Theralite^®^. As high-cutoff hemodialysis (HCO-HD) combined with chemotherapy has progressed over the last decade, questions have arisen regarding whether it can yield better clinical benefits than HF-HD and other conventional hemodialysis techniques for patients with AKI caused by MM and whether there is a target threshold of serum FLC reduction in these patients.

## Pathological features of AKI secondary to MM

**Figure 1:**
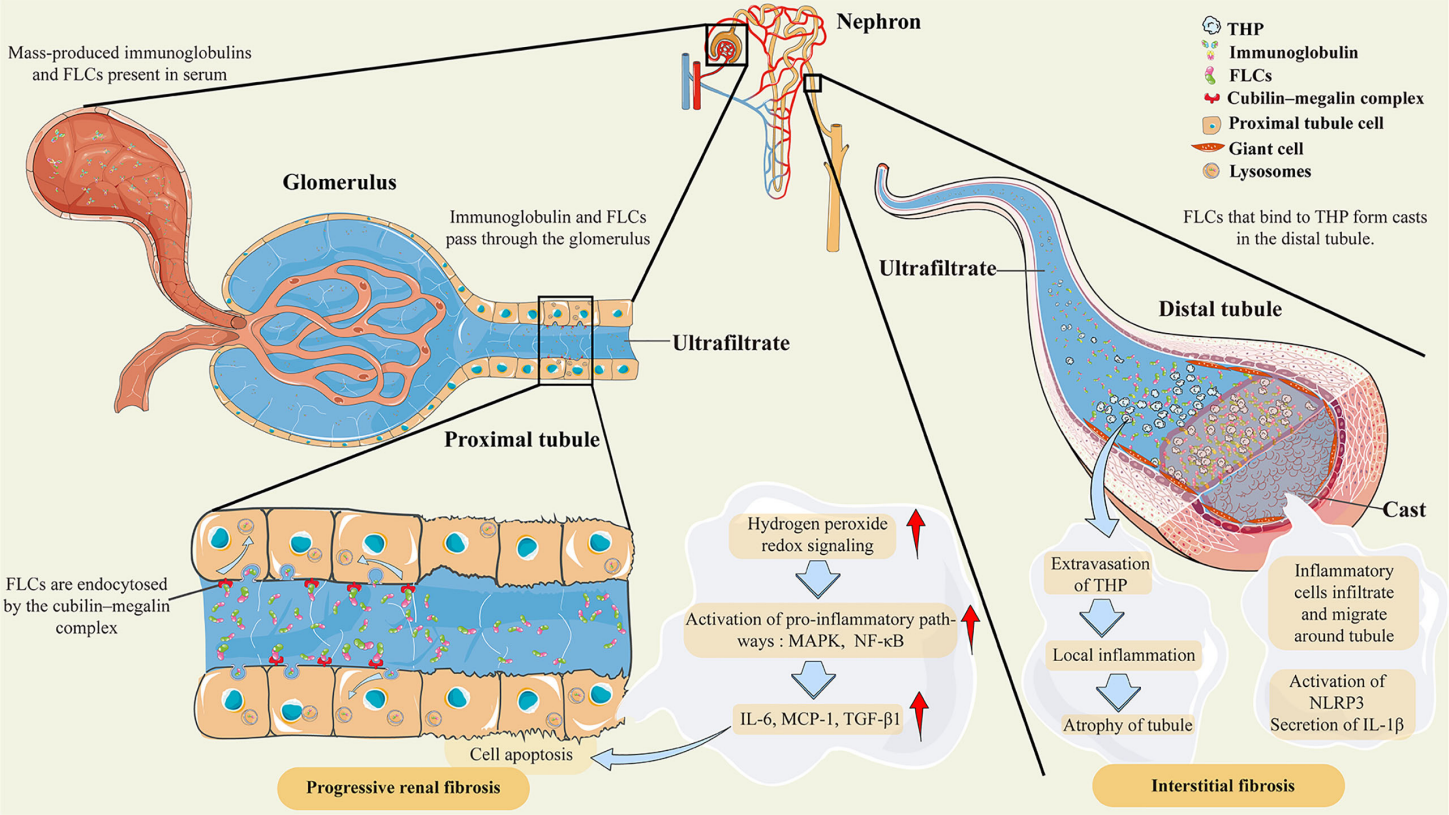
Pathological features of AKI secondary to multiple myeloma. Abbreviations: THP, Tamm–Horsfall protein; FLCs, free light chains; MAPK, mitogen-activated protein kinases; NF-κB, nuclear factor-κB; IL-6, interleukin-6; MCP-1, monocyte chemoattractant protein-1; TGF-β1, transforming growth factor β1; NLRP3, NOD-like receptor family protein 3; IL-1β, interleukin-1β. Under normal conditions, FLCs are endocytosed by the cubilin–megalin complex in the proximal tubule cell. Almost all FLCs will ultimately degrade in lysosomes. However, mass-produced immunoglobulins and FLCs are present in the serum as a result of clonal proliferation of plasma cells, exceeding the renal tubular reabsorption capacity. The massive amount of FLCs will induce hydrogen peroxide and redox signaling, leading to the activation of proinflammatory pathways, such as MAPK and NF-κB. Then, the transcription of inflammatory and profibrotic cytokines, such as IL-6 and MCP-1, is initiated. In the distal tubule, FLCs can bind to Tamm–Horsfall protein to form casts that obstruct the tubules, which leads to tubular rupture and extravasation of Tamm–Horsfall protein. These effects lead to local inflammation, inflammatory cell infiltration, and migration around tubules, and even to atrophy of tubules. There are also postulations that the crystalline organization of casts participates in inflammation and giant cell reactions by activating NLRP3 and secreting IL-1β.

The major cause of AKI in patients with MM is an increased concentration of serum monoclonal FLCs, which are reabsorbed in the proximal tubules and degraded in the lysosomes of proximal tubular cells after passing through the glomerulus (27). The body can produce approximately 500 mg of polyclonal FLCs each day (4, 28); however, less than 10 mg of polyclonal FLCs can be excreted in the urine because of reabsorption (29). In MM and other diseases in which clonal proliferation of plasma cells leads to an increase in monoclonal FLCs, mass-produced FLCs eventually exceed the renal tubular reabsorption capacity (4), as shown in **Figure 1**. Massive reabsorption of FLCs also reduces the catabolic capacity of proximal tubular cells (30) and induces the generation of hydrogen peroxide and redox signaling (27), ultimately promoting the activation of multiple inflammatory response pathways, such as the nuclear factor kappa-B (NF-κB) pathway (31), which is essential for MM progression (32, 33). In the distal tubule, FLCs can bind to Tamm–Horsfall protein to form casts that obstruct the tubules, which leads to tubular rupture and extravasation of Tamm–Horsfall protein, resulting in tubulointerstitial nephritis (34) and even AKI in some severe cases (35). The combination of these effects eventually leads to impairment of renal function and even irreversible fibrosis.

## HCO-HD research and the removal of FLCs

The significant production of FLCs in serum leads to renal impairment, which, in turn, leads to a reduction in FLCs that were cleared by the kidney and an increase in serum FLC concentration. Attempts to remove serum FLCs through appropriate renal replacement therapies are therefore urgently needed. In recent years, there have been many debates regarding the use of appropriate extracorporeal techniques to accelerate serum FLC removal on the basis of effective chemotherapy. PE is the first method to directly remove serum FLCs. Serum FLCs constitute approximately 15%–20% of the total FLC volume, and PE with 3.5 L of plasma removes approximately 65% of serum FLCs. A model of FLCs in MM suggests that only 25% of the total FLC volume can be removed by PE within 3 weeks (36). However, due to the similar concentration of FLCs in the serum, extravascular compartment, and tissue edema fluid (37) and the short duration of each session of PE (23), the reduction of FLCs in plasma enabled their easy diffusion back into blood vessels from the outside. Therefore, the substantial rebound of FLCs concentration, resulted in the ineffectual removal of FLCs by PE. Moreover, PE also removes many essential proteins, such as intact immunoglobulins and blood coagulation factors (23). A randomized controlled trial also revealed that using PE has no significant clinical benefits (38), which indicated that new alternative treatments need to be explored.

As the κ and λ chains of FLCs weigh 22.5 and 45 kDa, respectively, it is challenging for routine HF-HD to remove them. Before the introduction of novel chemotherapeutic agents, less than 25% of patients with AKI secondary to MM requiring dialysis treatment could be dialysis-independent (39). In the era of new chemotherapeutic agents, the dialysis-independent rate among patients treated with routine intermittent hemodialysis was approximately 30% (40). With the development of dialysis membranes and dialysis modalities, the advent of the HCO membrane has made it possible to efficiently remove serum FLCs through extracorporeal techniques.

Hutchison et al. performed *in vivo* and *in vitro* studies to demonstrate that the HCO1100 dialyzer with an extended dialysis time could produce more effective FLC clearance than PE, with an FLC clearance rate of 10–40 ml/min (36). A model of FLCs in MM indicates that 90% of the total FLC volume can be removed by HCO-HD within 3 weeks, and renal recovery was observed in three out of five patients with AKI secondary to MM (36). In subsequent research, it was discovered that raising the ultrafiltration rate increased the κ chain removal rate and that changing dialyzers in extended dialysis procedures to reduce membrane passivation could increase the λ chain removal rate (41). A 60% reduction in FLCs within 21 days restores renal function in 80% of patients with AKI secondary to MM (42).

Many studies have reported the removal rate of FLCs by HCO-HD. Specifically, Zannetti et al. observed that three 4-h weekly sessions of HCO-HD prior to a bortezomib-based regimen resulted in a 27% reduction in the difference between involved and uninvolved serum FLC (dFLC) concentrations and a significant increase in that difference after drug administration (43). Peters et al. performed a 5-h dialysis session combined with chemotherapy and observed that the use of a session of HCO-HD removed approximately 61 ± 20% of FLCs (44). In MYRE with a 5-h dialysis session, the median reduction rate of FLCs after the first HCO-HD was 68% (40). Berni et al. performed a 6-h session of HCO-HD in combination with chemotherapy, which resulted in a 40.2%–75.4% decrease in FLCs per session (45). Steiner et al. observed an average of 66.5 ± 20.88% FLC elimination with a session of HCO-HD in a real-world study but also a 35% rebound in FLC concentration following HCO-HD (46). In EuLITE (39), the median reduction rates of the κ and λ chains after an 8-h dialysis session combined with chemotherapy were 77% and 72%, respectively. Those abovementioned studies preliminarily indicated that the clearance of FLCs will increase with the extension of dialysis time and the addition of effective chemotherapy.

Several studies revealed that 41.7%–85.7% of patients recovered renal function and became hemodialysis-independent of HCO-HD, as summarized in **Table 1** (43–51, 53, 54). However, due to the inherent nature of retrospective studies, confounding factors, such as whether patients had a history of chronic kidney disease, hypercalcemia, and other precipitating factors of cast nephropathy, the percentage of atypical plasma cells, small sample sizes, different estimated glomerular filtration rates (eGFR) as end points for hemodialysis independence, and inconsistent follow-up times, were present across studies. Furthermore, not all patients in those studies had undergone a biopsy. Low urinary albumin with high serum FLC levels (>500 mg/L) is a warning sign for MCN in MM patients (4). **Table 1** also demonstrates that a large percentage of individuals who had a biopsy had MCN. Therefore, even without biopsy information, it appears reasonable that MCN is the most likely cause of AKI secondary to MM. However, we remain convinced that biopsy is a reliable way to assess renal prognosis and guide treatment because other monoclonal immunoglobulin-related kidney lesions, such as light-chain amyloidosis and light-chain deposition disease, are also potential reasons for AKI in MM patients. For patients with suspected MCN and serum FLC levels less than 500 mg/L, renal biopsy should be conducted without any contraindications (4, 55, 56).

**Table 1:** The renal recovery rate at the end point of previous studies.

Reference	Design	Sample size	Age (years)	Male (%)	Follow-up (months)	*De novo* MM (%)	FLC concentration at baseline (mg/L)	Serum creatinine at baseline (μmol/L)	MCN in biopsy patients ^b^	Renal recovery rate (%)
Hutchison et al., 2009 (47)	Prospective/ Pilot study/ Single-center	19	60	74	NA ^a^	84.2	2,600	714	19/19	73.7
Hutchison et al., 2012 (48)	Multi-center	67	65.1	62.7	NA	74.6	5,770	662	33/38	62.6
Heyne et al., 2012 (49)	Retrospective/ Single-center	19	69	36.8	NA	52.6	8,580	601.13	6/6	73.7
Sinisalo et al., 2012 (50)	Single-center	7	64	28.6	NA	100	13,300	589	6/6	85.7
Tan et al., 2014 (51)	Retrospective/ Multi-center	6	66	NA	7.5	83.3	12,500	651	6/6	50
Zannetti et al., 2015 (43)	Prospective/ Single-center	21	62	57.1	17.2	100	6,040	569.3	15/15	76
Berni et al., 2016 (45)	Single-center	13	63	76.9	NA ^a^	NA	7,110	565.8	5/8	76.9
Steiner et al., 2021 (46)	Retrospective/ Multi-center	61	66	68.9	NA ^a^	100	7,883	530.4	NA	42.6 at 3 months 49.2 at 6 months
Marn et al., 2016 (52)	Retrospective/ Single-center	28	68	53.6	NA	75	κ 14,433 λ 10,307	552	NA	60.7 ^c^
Peters et al., 2011 (44)	Prospective/ Case–control/ Single-center	HCO-HD 5 HD 5	HCO-HD 67.8 HD 74.2	HCO-HD 80 HD 20	HCO-HD 12.9 HD 13.3	NA	HCO-HD 2,528 HD NA	HCO-HD 660 HD 457.8	10/10	HCO-HD 60 HD 0
Gerth et al., 2016 (53)	Retrospective/ Case–control/ Single-center	HCO-HD 42 HD 17	HCO-HD 64.4 HD 58.4	HCO-HD 47.6 HD 58.8	NA ^a^	54.2	HCO-HD κ 8,545 λ 5250 HD κ 5,230 λ 12,400	HCO-HD 388.97 HD 442.01	16/17	HCO-HD 64.3 HD 29.4
Curti et al., 2016 (54)	Retrospective/ Cohort/ Multi-center	HCO-HD 12 HDF 7	HCO-HD 62.5 HDF 63.9	HCO-HD 83 HDF 57	HCO-HD 27.2 HDF 26.5	73.7	HCO-HD 11,924 HDF 8,043	NA	10/10	HCO-HD 41.7/58.3 at 3 months/end point HDF 16.7/33.3 at 3 months/end point

NA, not available or could not obtain all of the participants’ detailed information; FLCs, free light chains; MCN, myeloma cast nephropathy; HCO-HD, high-cutoff hemodialysis; HDF, hemodiafiltration.

Age, follow-up, FLC concentration, and serum creatinine are presented as the mean or median.

Renal recovery is defined as hemodialysis independence with no other extracorporeal techniques needed.

a Data represent the follow-up period, which lasted more than 3 months.

b The denominator represents the number of patients who underwent a biopsy, and the numerator represents the number of MCN patients.

c Hemodiafiltration was applied to the treatment.

To date, only two randomized controlled trials (RCTs), the EuLITE (39) and MYRE (40) experiments, have been performed to confirm the differences between HCO-HD and routine HF-HD. The major results are listed in **Table 2**. The results showed that HCO-HD does have a hemodialysis-independent rate of approximately 60% at 12 months, which was an improvement over the previous 30% renal recovery rate. However, neither of the two RCTs showed a statistically significant dialysis-independent rate in the first 3 months compared with the control group, although MYRE had a higher hemodialysis independence rate at 6 and 12 months than the control group. This may indicate that the recovery of kidney function takes a longer time in these patients. However, in EuLITE, there was no higher hemodialysis-independent rate and no better trend in the HCO-HD group. At 3 months, the MYRE showed a good rate of hematological response (partial response, very good partial response, and complete response) in the HCO-HD group because of the different methods of hemodialysis (*p* < 0.01). However, at 6 months, the hematological response was not statistically significant in either RCT. At 12 months, the EuLITE even showed that the hematological response of the HF-HD group was superior to that of the HCO-HD group (*p* < 0.05). EuLITE finally concluded that HCO-HD did not yield any practical benefits over HF-HD and was even associated with lower hematological remission at 12 months and higher mortality at the end point of follow-up than the HF-HD group.

**Table 2:** The major results of the two randomized controlled trials.

Groups	EuLITE (39)		MYRE (40)	
	HCO-HD	HF-HD	HCO-HD	HF-HD
Patients	43	47	46 ^a^	48
Study period	2008–2013		2011–2016	
Area	UK and Germany		France	
Age (years)	66	65	68	69
Serum creatinine (μmol/L)	623	499	566	645
Previous kidney disease (%)	7	2	6 ^b^	17 ^b^
Serum FLCs concentration (mg/L)	κ 9,300 λ 7,200	κ 11,600 λ 7,200	6590	5230
Bone marrow plasma cells (%)	NA	NA	38	31
Albumin (g/L)	37	38	32	34
First-line chemotherapy	BAD		BD	
Follow-up (months)	24		17.5 ^c^	
Hemodialysis independence rate at 3 months (%)	56	51	41	33
Myeloma response rate at 3 months (%)	NA	NA	89	63
Hemodialysis independence rate at 6 months (%)	58	66	57	35
Myeloma response rate at 6 months (%)	63	72	78	60
Hemodialysis independence rate at 12 months (%)	58	66	61	38
Myeloma response rate at 12 months (%)	42	68	NA	NA
Death rate at 12 months (%)	NA	NA	20	21
Death rate at end point (%)	37	19	28	33

NA, not available or could not obtain all of the participants’ detailed information; HCO-HD, high-cutoff hemodialysis; HF-HD, high-flux hemodialysis; FLCs, free light chains; BD, bortezomib and dexamethasone; BAD, bortezomib, doxorubicin, and dexamethasone.

Age, serum creatinine, serum FLC concentration, bone marrow plasma cells, and albumin are presented as the mean or median.

Hemodialysis independence is defined as sustained renal recovery without extracorporeal techniques after treatment.

a Data show that 46 patients were included in the primary analysis.

b Data represent patients with previous kidney diseases with an estimated glomerular filtration rate greater than 30 ml/min/1.73 m^2^.

c Data represent a median follow-up of 17.5 months (interquartile range, 12.0–30.0 months).

There were also certain differences between the two RCTs, such as whether medication to correct unfavorable variables was administered before randomization and differences in filters, chemotherapy regimens, and dialysis protocols. Because of the above differences, it has also been postulated that intensive HCO-HD is harmful in patients who can achieve renal response by steroids and symptomatic measures (57). However, determining the accurate cause of such opposing outcomes is challenging, as hemodialysis or chemotherapy cannot be individually administered to these patients. Thus, more trials based on uniform rules are needed and expected in the future, such as consistent chemotherapy regimens, to clarify the accurate reasons for this difference. The results of the only two RCTs placed serious doubt on the use of HCO-HD, yet both trials revealed that the HCO membrane combined with chemotherapy treatment exhibited strong clearance qualities for serum FLCs. The high clearance properties of HCO-HD versus HF-HD for FLCs and the higher hematological response at 3 months were also confirmed in a meta-analysis incorporating the results of these two RCTs and three cohort studies (44, 53, 54). Nevertheless, no significant difference was found between HCO-HD and HF-HD in terms of improving renal recovery outcomes (58). In another study of 83 patients with AKI secondary to MM, in which 31 patients needed dialysis, bortezomib-based triplets were associated with a high potential for kidney response (59). Among the 31 patients with routine hemodialysis and 70% who received a triplet, 15 patients ultimately became hemodialysis-independent. In EuLITE, where a triplet regimen based on bortezomib was used as the first-line treatment, but MYRE used a doublet, hemodialysis independence rates were also shown to be approximately 50% in the HF-HD group at 3 months and even 66% at 12 months. This may indicate that a combination of multiple chemotherapy drugs can make a significant difference in treatment. A phase II trial combining quadruple medication therapy with routine hemodialysis is now ongoing (46), which is expected to provide an alternative treatment for patients with AKI secondary to MM. As we mentioned above, the prevention of FLC formation is the primary goal. However, combining multiple medications may make critically ill patients more vulnerable to complications and even death. Trials with novel drugs such as monoclonal antibodies without the addition of more drugs are also needed for patients in the future.

## Adverse effects associated with HCO-HD

No serious complications associated with dialysis occurred during HCO-HD (36, 45–47, 50, 51, 60), and major reported adverse effects were almost always attributable to chemotherapy. The side effects related to hemodialysis included hypotension, fever, infections, muscle weakness, and thrombosis in the catheter. However, in two RCTs (39, 40), there were some dialysis-related adverse effects. The MYRE revealed no statistically significant dialysis-related complications in HCO-HD compared with HF-HD, demonstrating that HCO-HD is as safe as HF-HD, while there is a trend toward more adverse effects in HCO-HD (40). In EuLITE (39), 364 adverse events were recorded, with 52% in HCO-HD, and 54% of the serious adverse effects with grades 3–5 occurred in HCO-HD. The EuLITE trial finding that 8-h hemodialysis combined with triplet therapy resulted in a greater incidence of infection and mortality may demonstrate that HCO-HD should be adopted in a relatively mild manner rather than using a dialysis duration that is too long once combined with a triplet regimen.

Because of the high-molecular-weight cutoff of the HCO membrane, HCO-HD can cause considerable albumin loss. The amount of albumin loss decreased rapidly in the dialysis procedure, probably due to the blockage of the filter membrane (61). The loss of albumin can be avoided by proactive albumin supplementation during the last hour of dialysis. Hutchison et al. demonstrated that HCO-HD will result in the loss of 1.5 g and 5.7 g of albumin per hour in a single HCO1100 and a series of HCO1100, and at least 12 g and 45 g of albumin will be needed for 8-h HCO-HD, respectively (41). Twenty grams of albumin was frequently supplied after HCO-HD with Theralite^®^ (40, 45). During HCO-HD, the loss of electrolytes, such as calcium and magnesium, is common (47, 62).

## Protocols for HCO-HD

Since there is no consensus regarding the HCO-HD protocol, it is often left to the physician’s discretion. The protocols of the previous HCO-HD are provided in **Table 3**. The timing of HCO-HD initiation was not mentioned in previous studies. However, it is critical that HCO-HD should be implemented as soon as possible once AKI patients who need dialysis are diagnosed. A session is usually conducted intermittently for durations longer than 4 h. A serum FLC concentration of 500 mg/L has been set as the threshold for myeloma cast nephropathy (63). Thus, most studies terminated HCO-HD when the serum FLC concentration dropped below 500 mg/L (45, 49, 50, 54) and kept using HF-HD if dialysis was still needed until the eGFR was more than 15 ml/min/1.73 m^2^. Blood and dialysate flow were set to 250–350 and 500 ml/min, respectively, with ultrafiltration performed according to clinical needs. There is also a study that applied HCO membranes in post-dilution hemodiafiltration in MM patients with AKI (52).

**Table 3:** The protocols of previous high-cutoff hemodialysis or high-cutoff hemodiafiltration.

Reference	Design	eGFR (ml/min/1.73 m^2^)	Dialysis Membrane area (m^2^)	Hemodialysis procedure	Hemodialysis end point	Albumin supplement	Dialysis-related adverse effect	Hemodialysis sessions/days in patients became independent of dialysis
Hutchison et al., 2009 (47)	Prospective/ Pilot study/ Single-center	7	1.1 × 2	8 h/session daily for 5 days and on alternate days for next 12 days, then three 6 h/session per week	Until the patients became independent of dialysis	40 g after 8 h of dialysis	0	28 d ^h^
Hutchison et al., 2012 (48)	Multi-center	NA	1.1/1.1 × 2	2–4 h/4-6 h/>6 h ^c^	NA	NA	6% patients happened relevant side effect ^f^	11.5 ^i^
Heyne et al., 2012 (49)	Retrospective/ Single-center	7	1.1	6 h/session, five sessions in the first week, followed by every other day	sFLC concentrations < 500 mg/L	NA	NA	15 d ^h^ 6 ^i^
Sinisalo et al., 2012 (50)	Single-center	8	2.1	3 h low-flux hemodialysis at first session, then daily 6 h/session	sFLC concentrations < 500 mg/L	NA	0	17.5 ^i^
Tan et al., 2014 (51)	Retrospective/ Multi-center	8	1.1/1.1 × 2/2.1	NA	NA	NA	0	6 ^i^
Zannetti et al., 2015 (43)	Prospective/ Single-center	8 ^a^	1.1	4 h/session, thrice weekly	The reduction of sFLC concentrations > 60%	NA	NA	32 d ^h^
Berni et al., 2016 (45)	Single-center	9	2.1	6 h/session daily for 6 days and then 6 h/session on alternate days	sFLC concentrations < 500 mg/L or renal recovery	20 g in the last hour of dialysis	0	NA
Steiner et al., 2021 (46)	Retrospective/ Multi-center	7 ^a^	NA	NA	NA	NA	0	11 ^i^
Marn et al., 2016 (52)	Retrospective/ Single-center	NA	2.1	8 h/session daily or every other day ^d^	sFLC concentrations < 500 mg/L or renal recovery	40 g in the last 2 h of dialysis	0	NA
Peters et al., 2011 (44)	Prospective/ Case–control/ Single-center	NA	1.1	5 h/session, 6 days/week for 6 weeks	It depended on patients’ tolerance or creatinine clearance > 15 ml/min	20 g in the last hour of dialysis	NA	41 ^i^
Gerth et al., 2016 (53)	Retrospective/ Case–control/ Single-center	NA ^a^	1.1 × N ^b^/2.1	About 6 h/session and at least 5 sessions per week	sFLC concentrations < 1,000 mg/L	NA	NA	NA
Curti et al., 2016 (54)	Retrospective/ Cohort/ Multi-center	7.7	1.1/2.1	8 h/session daily for 5 days and on alternate days for next 12 days, then 6 h/session thrice weekly	sFLC concentrations < 500 mg/L	NA	NA	NA
Bridoux et al., 2017 (40)	Randomized controlled trial	NA	2.1	Eight 5 h/session for the first 10 days. If needed, 3 additional weekly hemodialysis sessions until completion of 3 cycles of chemotherapy	Individual investigators determined hemodialysis withdrawal	20 g if albumin less than 25 g/L prior to dialysis	Incidence of dialysis-related effect is 43% ^g^	NA
Hutchison et al., 2019 (39)	Randomized controlled trial	7	1.1 x 2	6 h/session on day 0, then 8 h/session on day 2, 3, 5–7, 9, 10. After day 12, 8 h/session on alternate days and from day 21, 6 h/session thrice weekly up to 90 days	Nephrologist determined hemodialysis withdrawal ^e^	60 g in the last hour of dialysis	NA	NA

NA, not available or could not obtain all of the participants’ detailed information; eGFR, estimated glomerular filtration rate, presented as the mean or median; sFLC, serum free light chain.

a Data are calculated by the CKD-EPI (Chronic Kidney Disease Epidemiology Collaboration) equation.

b Data indicate the 1.1-m^2^ dialysis membrane used in single or series.

c Data indicate the duration of hemodialysis (2–4 h, 4–6 h, or>6 h) without detailed protocol information.

d Hemodiafiltration was applied to the treatment.

e Patients with a predialysis eGFR of more than 20 ml/min/1.73 m^2^ and an adequate urine output were advised to stop dialysis.

f Dialysis-related adverse effects, including hypotension, fever with negative culture, reversible muscle weakness, and thrombosis of the central venous catheter, occurred in 6% of patients.

g Rate of hemodialysis-related adverse events of any grade was 43% in HCO-HD.

h Data presented as the median number of days until dialysis independence was achieved.

i Data presented as the median number of sessions in patients who became independent of dialysis.

## Other extracorporeal techniques can be available to achieve a reduction in FLCs

Although the reduction of FLCs is not as significant as HCO-HD, researchers also reported other extracorporeal techniques that may have beneficial effects on patients with AKI secondary to MM. The removal efficacy of FLCs by different extracorporeal techniques is demonstrated in **Table 4**.

**Table 4:** Other extracorporeal techniques can be available to reduce FLCs.

Reference	Design	Sample size	Age (years)	Male (%)	Extracorporeal techniques	FLC concentration at baseline (mg/L)	Serum creatinine at baseline (μmol/L)	Hemodialysis procedure	Reduction of κ FLC	Reduction of λ FLC
Santoro et al., 2013 (64)	Pilot study/ Single-center	7	77	14.3	PMMA	3,648	NA	4 h/session	1 PMMA ^a^ 27.7% 2 PMMA ^b^ 33.1%	1 PMMA ^a^ 15.2% 2 PMMA ^b^53.1%
Fabbrini et al., 2013 (26)	Retrospective/cohort/ Multi-center	10	NA	NA	PMMA	1 PMMA 6,538 2 PMMA 7,925	NA	4 h/session	1 PMMA ^a^ 22.3% 2 PMMA ^b^ 31.0%	1 PMMA ^a^ 21.0% 2 PMMA ^b^ 53.1%
Sens et al., 2017 (65)	Retrospective/cohort/ Single-center	17	62	47.1	PMMA	4,220	485	Six 6-h sessions a week for a maximum of 3 weeks	NA	NA
Pendón-Ruiz et al., 2013 (66)	Single-center	3	63	33.3	Supra-HFR	κ 1,873.6 λ 160.4	NA	2 cases with three 4-h sessions a week and 1 case initially with 3-h session a day then a progressive reduction of sessions	53.8%–63.4% per session ^c^	38% per session ^c^
Pasquali et al., 2015 (24)	Single-center	4	63	25	Supra-HFR	10,145	715.7	4 h/session for 8 consecutive days and then every 2 days	4.9–15.3ml/min ^c^	3.2–11.5 ml/min ^c^
Menè et al., 2018 (67)	Pilot study/ Single-center	6	61	50	Supra-HFR	6,910	972.4	5 consecutive sessions on alternate days, the first 2 sessions lasted 3 h and 4 h for the rest of 3 sessions	84.01%	69.3%
Li et al., 2018 (68)	Single-center	3	NA	NA	Supra-HFR	1,130	NA	Three 3.5-h sessions a week	NA	32.2%–49.5%
Pendón-Ruiz et al., 2020 (69)	Observational/Single-center	9	72	44.4	Supra-HFR	κ 11,200 λ 1,313	750	The first two sessions last 2.5 h and 3 h, respectively; the remaining sessions last 4 h three times a week	57.6%	33.5%
Cazorla et al., 2020 (70)	Single-center	3	72	66.7	MCO-HD	14,300	415.5	6 h/session	44.8% per session ^c^	NA

Abbreviations: NA, not available or could not obtain all of the participants’ detailed information; FLCs, free light chains; PMMA, polymethylmethacrylate; Supra-HFR, hemodiafiltration with ultrafiltrate regeneration by adsorption in resin; MCO-HD, medium-cutoff hemodialysis.

Age, FLC concentration, and serum creatinine are presented as the mean or median.

a Data show that only one PMMA membrane was adopted in hemodialysis.

b Data show replacement of the PMMA membrane at the midpoint of hemodialysis.

c Data present the trend of FLC reduction.

### Hemodialysis with an adsorbent polymethylmethacrylate membrane

The PMMA membrane is highly biocompatible and can be used in standard HD. If the PMMA membrane is replaced halfway through the session, the reduction in FLCs will be increased. Santoro et al. (64) observed a 27.7% reduction for the κ chain and 15.2% for the λ chain by using a PMMA membrane in 4-h routine hemodialysis sessions from the beginning to the end of treatment. However, the reduction increased to 33.1% and 53.1% for the κ and λ chains, respectively, when two PMMA membranes were adopted. In another study using PMMA membranes for 4-h routine hemodialysis sessions in dialysis-dependent patients with serum FLCs above 500 mg/L, the reduction rates of κ and λ chains were only 22.3% and 21%. respectively, but increased to 31% and 53.1% after changing the PMMA membrane halfway through the session (26). Briefly, two PMMA membranes may be an option for patients with a high-concentration λ chain. In a series of 17 patients with AKI secondary to MM requiring dialysis (65), 88% of patients received a bortezomib-based chemotherapy regimen combined with six 6-h hemodialysis sessions a week with two PMMA membranes until the serum FLCs dropped below 200 mg/L. Twelve (70.6%) patients recovered renal function in 60 days. Among the 14 patients who could be evaluated for hematological response, 7 patients achieved a very good hematological response or better at 3 months and did not require albumin supplementation during treatment. The dialyzer was replaced midway through the study due to the adsorption saturation properties of the PMMA membrane, which indicated the effectiveness of the PMMA membrane.

### Hemodiafiltration with ultrafiltrate regeneration by adsorption in resin

**Figure 2:**
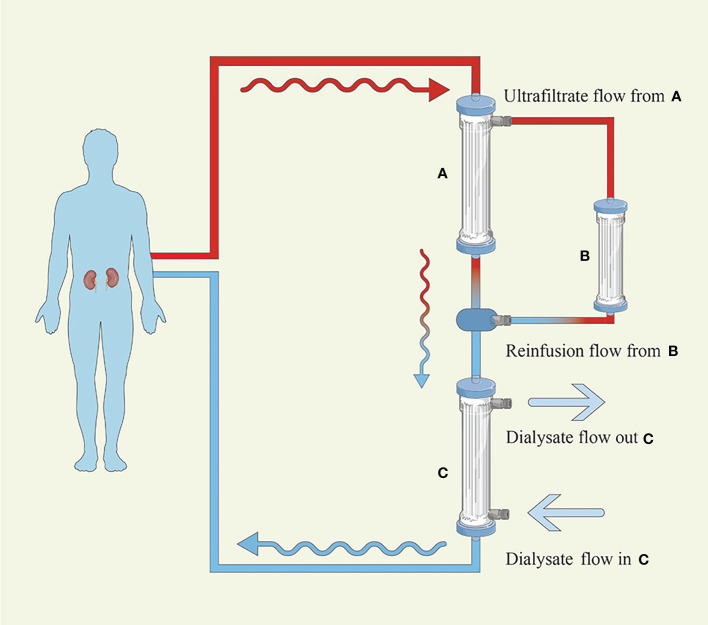
Hemodiafiltration with ultrafiltrate regeneration by adsorption in resin. **(A)** High-permeability filter with a polyphenylene membrane surface of 0.7 m^2^ **(B)** Resin cartridge with a high affinity for κ and λ **(C)** Low-permeability filter with a polyphenylene membrane surface of 1.7 m^2^. The blood flows into the A filter initially, where convection is performed, and the produced ultrafiltrate transits the B cartridge, where adsorption takes place, before passing through the C filter, where diffusion is carried out.

SUPRA-HFR utilizes convection, adsorption, and diffusion by connecting a high-permeability filter to a low-permeability filter and making the ultrafiltrate from a high-permeability filter pass through a resin cartridge followed by reinfusing it into a low permeability filter. The process is shown in **Figure 2**. The resin cartridge plays a major role in reducing FLCs. SUPRA-HFR achieves effective removal of FLCs by resin and avoids the loss of albumin, which was verified in some small-sample observation studies (24, 66–69). Pasquali et al. (24) observed a significant decrease in FLCs, and three out of four patients with AKI secondary to MM became dialysis-independent in 6 weeks through SUPRA-HFR combined with bortezomib-based chemotherapy. They also found a reduction rate of 4.9–15.3 ml/min and 3.2–11.5 ml/min for the κ chain and λ chain, respectively. However, it was also found in another study that only 3 out of 9 MM patients with AKI became dialysis-independent after 2.75 ± 0.43 months of treatment (69). These two studies all show that the efficiency of the resin cartridge decreases over time, especially for the λ chain. The efficiency of the κ chain decrease is greater than that of the λ chain in SUPRA-HFR, which has also been observed in other studies (24, 66, 71). The total reduction of the κ chain was also better than that of the λ chain (67, 69). Therefore, it is recommended that SUPRA-HFR should be applied in patients with a high concentration of the κ chain (66).

### Medium-cutoff hemodialysis

The MCO membrane has a relatively smaller pore size than the HCO membrane, which also induces a moderate loss of albumin and is more commonly used in patients requiring maintenance hemodialysis. Medium-cutoff hemodialysis (MCO-HD) has been confirmed to be superior to HF-HD and hemodiafiltration (72, 73). A crossover multicenter clinical trial also confirmed that MCO-HD in patients with end-stage kidney disease for 6 months is safe, with no substantial drop in albumin and no influence on quality of life, functional status, or nutrition (74). However, research on the use of MCO-HD for AKI secondary to MM is limited. In a series of three patients with AKI secondary to MM requiring dialysis treatment due to an elevated concentration of κ chain, MCO-HD combined with chemotherapy induced a sustained decrease in the κ chain with an average decrease of 44.8 ± 19.5% per session. Eventually, all three patients became dialysis-independent (70).

As mentioned above, two PMMA membranes can reduce the λ chain by more than 50% and the κ chain by 30%. SUPRA-HFR has the ability to reduce more κ chains than λ chains. The MCO-HD for AKI secondary to MM was limited. However, at least an average 44.8% reduction for the κ chain was available. Their adverse effects are less than those of HCO-HD, but the efficiency of FLC reduction is also inferior to that of HCO-HD.

## What we should know

In recent years, extracorporeal techniques have made great progress in the removal of larger middle molecules. Nevertheless, it is not sufficient to simply speed up the clearance of FLCs in patients with AKI secondary to MM. It is crucial to use effective chemotherapeutic medications to control the production of FLCs. Proteasome inhibitors such as bortezomib have been proven to be associated with a better and faster renal response due to their effect on rapidly reducing FLCs through the NF-κB pathway (75). More importantly, bortezomib can be used without dose adaptation in patients with severe renal impairment, especially those requiring dialysis treatment (76, 77).

For patients with AKI secondary to MM who require dialysis support, hemodialysis and chemotherapy cannot be used separately. The duration of AKI prior to treatment initiation has been found to be an independent predictor of renal recovery (48, 49). However, the key predictor of renal recovery is the initiation time of efficient treatment to reduce the amount of FLCs (47, 48, 59). Therefore, there is an urgent need for a strong working relationship between nephrologists and hematologists. There is no doubt that the timely adoption of HCO-HD in conjunction with antimyeloma treatment in patients who require dialysis support is important. However, starting HCO-HD without effective chemotherapy would have limited benefits in MM patients with AKI. The use of HCO-HD alone does not effectively reduce the total amount of FLCs because the generation of FLCs is still ongoing. Additionally, FLCs easily diffuse back into blood vessels from the extravascular compartment or tissue edema fluid. It is critical to reduce the formation of serum FLCs through effective chemotherapy. Bortezomib-based chemotherapy is currently recommended for MM patients with AKI. Triplet chemotherapy regimens should be considered in fit patients (4); however, the best drug combination is still unclear, and there are tolerance concerns (4, 39). In critical patients, bortezomib and dexamethasone doublets should be considered first (4). It should also be noted that monoclonal anti-CD38 antibody, which has proven its rapidity and depth of hematological responses in MM patients (78, 79), is becoming increasingly more considerable in MM patients with AKI who need dialysis support (80). However, more trials are needed in the future.

Finally, there is no adequate evidence that HCO-HD should be the standard of care for all MM patients with AKI. MM patients with AKI who do not require dialysis support may recover renal function if the reversible factors can be corrected by volume expansion, urine alkalinization, blood calcium reduction, avoidance of nephrotoxic drugs, and initiation of chemotherapy. Hemodialysis should be performed as soon as possible when someone has indications for dialysis, such as severe acute kidney injury (AKI stage 3[KDIGO]), electrolyte disorder (blood potassium elevated more than 6.5 mmol/L), or severe volume overload (18). The absence of standards for HCO-HD also results in a variety of dialysis durations. The target threshold of serum FLC reduction and the timing of HCO-HD initiation are still unclear. Whether HCO-HD is the most cost-effective option is also still under debate due to the increased cost of HCO filters, albumin supplementation, and careful monitoring of electrolytes. However, some studies concluded that the total cost of HCO dialyzers was comparable to those of standard dialyzers (54), and some even demonstrated significant cost savings (45). This difference may be due to the different reimbursement policies in different countries. Therefore, the promotion of HCO-HD also needs to take the actual situation into account in each country. Although HCO-HD and other extracorporeal removal techniques are successful in removing pathogenic materials, current clinical trials have not yet confirmed the obvious advantage in recovering kidney function in AKI secondary to MM.

## Conclusions

In summary, there are no ideal extracorporeal techniques that have emerged that can achieve large reductions for FLCs other than HCO-HD. HCO-HD and bortezomib-based chemotherapy have a tendency to improve renal outcomes, but more RCTs based on novel drugs are warranted. Perhaps HCO-HD should be used in MM patients with AKI who are unable to achieve restored renal function by steroids, symptomatic measures, and effective chemotherapy. For patients with high concentrations of κ or λ chains, there are other, less expensive extracorporeal techniques available even though the reduction of FLCs is inferior to HCO-HD, which also requires further investigation and validation.

## Author contributions

Conceptualization: YX, JY and SS. Validation: ZY and JZ. Supervision: YW, XL and YQ. Visualization: YX and JY. Writing—original draft: YX. Writing—review and editing: YX, JY, ZY and SS. All authors contributed to the article and approved the submitted version.

## Funding

This study was sponsored by grants from the Xijing Hospital Discipline Promoting Plan (Reference number: XJZT18MDT17) and National Natural Science Foundation of China grants (Reference number: 81870470).

## Acknowledgments

Figures were partly generated using Servier Medical Art, provided by Servier, licensed under a Creative Commons Attribution 3.0 unported license.

## Conflict of interest

The authors declare that the research was conducted in the absence of any commercial or financial relationships that could be construed as a potential conflict of interest.

## Publisher’s note

All claims expressed in this article are solely those of the authors and do not necessarily represent those of their affiliated organizations, or those of the publisher, the editors and the reviewers. Any product that may be evaluated in this article, or claim that may be made by its manufacturer, is not guaranteed or endorsed by the publisher.
